# gNODE: gLV model-informed neural ordinary differential equations for modeling microbial community dynamics

**DOI:** 10.3389/fcimb.2026.1785750

**Published:** 2026-07-06

**Authors:** Xiaoxiu Tan, Feng Xue, Lu Xie, Tao Wang

**Affiliations:** 1 Shanghai-MOST Key Laboratory of Health and Disease Genomics, Shanghai Institute for Biomedical and Pharmaceutical Technologies, Shanghai, China; 2 Department of Bioinformatics and Biostatistics, School of Life Sciences and Biotechnology, Shanghai Jiao Tong University, Shanghai, China; 3 Department of Statistics and MOE-LSC and CMA-Shanghai, School of Mathematical Sciences, Shanghai Jiao Tong University, Shanghai, China; 4 SJTU-Yale Joint Center for Biostatistics and Data Science, National Center for Translational Medicine, Shanghai Jiao Tong University, Shanghai, China

**Keywords:** dynamic modeling, microbiome, ordinary differential equations, species interactions, time-series data

## Abstract

**Background:**

The human gut microbiota is a highly complex ecological system closely linked to host health, yet the functional mechanisms underlying its dynamic behavior remain poorly understood. Accurate modeling of microbial community dynamics is essential for elucidating these mechanisms. However, most existing approaches rely on densely sampled time-series data and often lack biological interpretability.

**Methods:**

To address these challenges, we propose gNODE, a framework that integrates the generalized Lotka-Volterra (gLV) model with neural ordinary differential equations (NeuralODEs) to jointly predict microbial community dynamics, infer species interactions, and quantify the functional contributions of key taxa. By embedding ecological equations into a neural architecture, gNODE incorporates biological constraints directly into its model structure, enabling biologically meaningful parameter estimation and accurate inference even under sparse temporal sampling.

**Results:**

Through simulations and real datasets, gNODE demonstrates superior performance in parameter estimation, trajectory prediction, and perturbation response modeling compared with existing methods. In a *Clostridioides difficile* infection dataset, gNODE accurately captured post-infection community trajectories and identified key inhibitory taxa, highlighting its potential to discover microbes that suppress pathogens. In a probiotic cocktail colonization dataset, gNODE identified diet-specific keystone species, underscoring its utility for assessing perturbation responses and guiding the design of probiotic consortia.

**Conclusion:**

gNODE provides a robust and interpretable framework for modeling complex microbial community dynamics, offering new mechanistic and functional insights into the ecological processes that shape host-associated microbiomes.

## 1 Introduction

The human gut microbiome is a complex and dynamic ecosystem whose composition and function are shaped by diverse factors, including diet, medical interventions, and environmental exposures (Costello et al., 2012; Lozupone et al., 2012; Gerber, 2014). Microbiome variation occurs across the human life course, and its plasticity offers opportunities for microbiome-based therapies, particularly for preventing and treating diseases associated with dysbiosis (Lemon et al., 2012). Accurate prediction of microbial community dynamics is thus essential for identifying links between community disruption and disease and for guiding personalized interventions, such as dietary modification, probiotic administration, or fecal microbiota transplantation (FMT), aimed at restoring a healthy state.

Microbial community dynamics emerge from intricate species interactions; however, experimentally inferring these interactions remains challenging due to the high dimensionality of microbial systems and the difficulty of cultivating many taxa in isolation (Lidicker, 1979; Faust and Raes, 2012; Marino et al., 2014; Lagier et al., 2018; Culp and Goodman, 2023). With the rapid accumulation of large-scale sequencing datasets (The Human Microbiome Project Consortium, 2012a; The Human Microbiome Project Consortium, 2012b), co-occurrence networks have become widely used analytical tools, but they capture only statistical associations and cannot distinguish direct from indirect effects, thereby limiting their ability to reveal true ecological interactions (Liu et al., 2011; Agler et al., 2016; Ma et al., 2016; Röttjers and Faust, 2018). Moreover, cross-sectional datasets lack temporal information, posing additional challenges for elucidating the mechanistic interactions that govern community behavior (Stein et al., 2013).

Longitudinal studies of the human gut microbiome have become increasingly common in recent years, generating time-series data that enable deeper investigation of community dynamics, species interactions, and responses to environmental perturbations, thereby helping to address these limitations (Zhou et al., 2024). Concurrently, ecological dynamical models offer a principled framework for understanding microbial diversity and interspecies relationships (Xiao et al., 2020). Several studies have leveraged such models to infer microbial interactions from high-throughput data, reconstruct ecological networks and predict community trajectories (Bucci et al., 2016; Tan et al., 2024). Among these approaches, the generalized Lotka-Volterra (gLV) model, which is formulated as a system of ordinary differential equations (ODEs), has been widely applied to gut microbiome dynamics (Stein et al., 2013; Buffie et al., 2015). For example, extended gLV models have been applied to intestinal microbiome time-series data to infer ecological dynamical parameters and analyze community steady states and stability (Stein et al., 2013). More generally, because of its simple structure and ecologically interpretable parameters, the gLV model has become an important baseline framework for microbial interaction inference; however, reliable inference often depends on high-quality and sufficiently dense time-series data and is constrained by model assumptions and parameter identifiability (Davis et al., 2022).

Classical approaches for ODE parameter estimation, including gold-standard methods and two-step collocation schemes, have laid an important theoretical foundation for the practical application of ODE models (Chen et al., 2017). Gold-standard methods estimate parameters by minimizing the discrepancies between observed data and numerical ODE solutions, thereby yielding highly reliable estimates consistent with the assumed biological mechanisms; however, they become computationally prohibitive for high-dimensional systems (Benson, 1979; Biegler et al., 1986). To alleviate these computational challenges, many researchers have adopted two-step collocation approaches, which first fit smoothing functions to approximate the observed data and then use the smoothed estimates and their derivatives to infer the model parameters (Brunel, 2008; Liang and Wu, 2008; Chen et al., 2017). Although computationally more efficient, two-step collocation approaches critically rely on sufficiently dense data to obtain accurate smooth trajectories and derivative estimates. In addition, weak-form ODE parameter estimation methods have been developed to improve computational efficiency and robustness to measurement noise when the ODE model structure is known (Bortz et al., 2023). Taken together, these approaches highlight the continuing challenge of estimating interpretable dynamical parameters from longitudinal ecological data, particularly when temporal sampling is sparse and the number of parameters increases rapidly with community size.

As an alternative, deep learning approaches have been increasingly explored for modeling microbiome dynamics (Hernández Medina et al., 2022). In particular, neural ordinary differential equations (NeuralODEs) have been applied to ecological and evolutionary time-series analysis, providing flexible nonparametric tools for learning dynamical functions and inferring ecological interactions from time-series data (Bonnaffé et al., 2021). Subsequent work proposed Bayesian neural gradient matching to improve the fitting efficiency of ecological NeuralODE models by interpolating time series and their underlying dynamics with neural networks (Bonnaffé and Coulson, 2023). These studies demonstrate the potential of NeuralODEs for ecological dynamical modeling. However, existing NeuralODE-based approaches mainly emphasize flexible trajectory learning and nonparametric interaction inference, but do not directly address interpretable gLV parameter estimation under sparse longitudinal sampling. This motivates new dynamical modeling strategies that combine the ecological interpretability of mechanistic models with the flexibility of continuous-time neural learning.

To address these challenges, we propose gNODE, a gLV-informed NeuralODE framework that directly embeds ecological gLV dynamics into the NeuralODE architecture. Unlike black-box NeuralODEs that learn unconstrained dynamical functions, gNODE explicitly parameterizes species-specific growth rates and pairwise interaction coefficients, enabling interpretable ecological parameter estimation, trajectory prediction, and perturbation response analysis under sparse temporal sampling. Moreover, gNODE enables in silico perturbation experiments by systematically knocking out individual species and examining the resulting changes in community dynamics. Such structural perturbations facilitate the identification of mechanisms underlying community regulation and provide a quantitative assessment of species importance. Through simulation studies and analyses of two real datasets, including a *C. difficile* infection dataset and a probiotic cocktail colonization dataset, we demonstrate the advantages of gNODE in parameter estimation, trajectory prediction, and perturbation response analysis, and highlight its potential for identifying microbes that suppress pathogen growth and for evaluating perturbation-sensitive taxa.

## 2 Methods

### 2.1 Generalized Lotka-Volterra model

To characterize microbial community dynamics, we use the generalized Lotka-Volterra (gLV) model, a system of coupled ODEs that extends the classical predator-prey framework to multispecies communities (Mounier et al., 2008; Marino et al., 2014). Let $y_{ij}(t)$ denote the absolute abundance of species *j* in subject *i* at time *t*, and let $y_{ij}^{'}(t)$ represent its temporal derivative, where i=1,…,S represents the subject index and j=1,…,P represents species index. The gLV model is defined as:

(1) $$y_{ij}^{'}(t)=\alpha_{j}y_{ij}(t)+\sum_{k=1}^{P}\beta_{jk}y_{ij}(t)y_{ik}(t)$$

where $\alpha_{j}$ denotes the intrinsic growth rate of species *j*, and $\beta_{jk}$ quantifies the interaction strength of species *k* on species *j*, capturing competition, cooperation, or neutral ecological relationships among species.

### 2.2 Neural ordinary differential equations

In this study, we employ neural ordinary differential equations (NeuralODEs) as a general modeling framework (Chen et al., 2018). Unlike conventional neural networks, which represent transformations as a sequence of discrete hidden layers, NeuralODEs parameterize the time derivative of a continuous state variable using a neural network, thereby modeling its evolution in continuous time. Specifically, the dynamics of the state variable z(t) are given by:

(2) $$\frac{dz(t)}{dt}=f(z(t),t;\theta )$$

where θ denotes the set of trainable parameters.

In this study, the state variable of the NeuralODE is defined directly in the microbial abundance space. Thus, for subject *i*, the generic state $z_{i}(t)$ corresponds to the model-predicted microbial abundance trajectory, denoted by ${\hat{y}}_{i}(t;\theta )$. Given an initial condition $y_{i}(t_{0})={\left(y_{i1}(t_{0}),\ldots ,y_{iP}(t_{0})\right)}^{T}$ for subject *i*, the predicted abundance vector at any observation time point $t_{m\;}$(for m=0,…,T−1) can be obtained by solving the corresponding initial value problem using an ODE solver, yielding a continuous trajectory:

(3) $$\;\;{\hat{y}}_{i}(t_{m};\theta )=y_{i}(t_{0})+\int_{t_{0}}^{t_{m}}f({\hat{y}}_{i}(t;\theta ),t;\theta )dt=ODESolve(y_{i}(t_{0}),f,t_{0},t_{m},\theta )$$

In practice, we assume that the observations $y_{i}(t_{m})$ are subject to independent measurement error at *T* time points $\left\{t_{m},m=0,\ldots ,T-1\right\}$. To estimate the parameters θ, we minimize the discrepancy between the predicted trajectories and the observations:

(4) $$L_{data}(\theta )=\frac{1}{SPT}\sum_{i=1}^{S}\sum_{m=0}^{T-1}∥y_{i}(t_{m})-{\hat{y}}_{i}(t_{m};\theta ){∥}^{2}$$

Here, we use the $L_{2}$ loss to measure the data-fitting error.

### 2.3 gNODE: gLV-informed NeuralODEs

The primary goal of standard NeuralODEs is to fit and predict trajectories; they are not specifically designed for parameter estimation or model interpretability. Moreover, training NeuralODEs typically relies on relatively densely sampled time series; however, microbiome data are often sparsely sampled, and NeuralODE models generally do not incorporate explicit constraints that encode underlying ecological dynamics. These limitations motivate structured and interpretable NeuralODE frameworks tailored to sparse longitudinal microbiome data.

In ecological community modeling, the functional form of the governing equations (e.g., the gLV model) is often known, but the corresponding parameters must be inferred from data. Inspired by physics-informed neural networks (PINNs) (Karniadakis et al., 2021), we propose a new modeling approach, gLV-informed NeuralODEs (gNODE), in which the gLV equations are explicitly embedded as the dynamical system governing a NeuralODE, thereby enabling direct inference of ecological parameters from longitudinal observations.

Specifically, we construct a NeuralODE whose dynamics are governed by the gLV function $f_{\text{gLV}}({\hat{y}}_{i}(t;\theta );\theta )$, parameterized by $\theta =\{\alpha_{j},\beta_{jk}\}$. The dynamical system for gNODE is then given by:

(5) $$\frac{d{\hat{y}}_{i}(t;\theta )}{dt}=f_{\text{gLV}}({\hat{y}}_{i}(t;\theta );\theta )$$

Under this construction, by using the ODE solver, the data-fitting loss for gNODE can be written as:

(6) $$L(\theta )=\frac{1}{SPT}\sum_{i=1}^{S}\sum_{m=0}^{T-1}∥y_{i}(t_{m})-ODESolve(y_{i}(t_{0}),f_{\text{gLV}},t_{0},t_{m},\theta ){∥}^{2}\;\;$$

thus, the ecological parameters θ are optimized directly under the structural constraint imposed by the gLV function. Through parameter sharing in the differential equation, gNODE encodes species-specific intrinsic growth rates and pairwise interaction coefficients (i.e., the ecological network) explicitly into the neural parameter space, thereby jointly optimizing mechanistic ecological structure and data-driven dynamics. Compared with standard NeuralODEs that learn an unconstrained black-box dynamical function, gNODE provides improved interpretability, ecological plausibility, and robustness under sparse temporal sampling, offering an interpretable approach for dynamical modeling of microbiome communities.

### 2.4 pNODE: physics-informed NeuralODEs

As a baseline, we implemented a physics-informed NeuralODE (pNODE) following the PINN formulation. pNODE augments a standard NeuralODE by introducing a residual penalty derived from the gLV equations, thereby incorporating both data-fitting information and ecological structure.

The loss function for pNODE consists of two components. The first is a data-fitting term $L_{data}(\theta )$ that measures the discrepancy between model predictions and observations, as defined in Equation 4. The second component is a gLV residual loss. We uniformly sample time points $\tau_{m}$ over the time interval with step size 1 (with M=25,m=1,…,M) and minimize the squared residuals to encourage the predicted dynamics to satisfy the gLV equation:

(7) $$L_{ODE}(\theta )=\frac{1}{SPM}\sum_{i=1}^{S}\sum_{j=1}^{P}\sum_{m=1}^{M}\;{\left({\hat{y}}_{ij}^{'}(\tau_{m};\theta )-\alpha_{j}{\hat{y}}_{ij}(\tau_{m};\theta )-\sum_{k=1}^{P}\beta_{jk}{\hat{y}}_{ij}(\tau_{m};\theta ){\hat{y}}_{ik}(\tau_{m};\theta )\right)}^{2}$$

where ${\hat{y}}_{ij}^{'}(\tau_{m};\theta )$ is the temporal derivative of the predicted abundance given by the NeuralODE dynamical function.

The total loss is defined as a weighted sum of the two components, with weights $w_{\text{data}}$ and $w_{\text{ODE}}$, respectively:

(8) $$L=w_{data}L_{data}(\theta )+w_{ODE}L_{ODE}(\theta )$$

By minimizing this combined loss, pNODE integrates data-driven learning with dynamical constraints. Unlike gNODE, pNODE does not embed the gLV equations directly into the NeuralODE architecture; instead, these dynamics are enforced via a residual penalty. The implementation structures of gNODE and pNODE are illustrated in **Supplementary Figure 1**.

### 2.5 Model training

For model construction, we implemented the training of NODE, gNODE, and pNODE using the *torchdiffeq* library in *PyTorch*. Data loading and data organization were performed via a custom *Dataset* class. Model training was performed at the subject-trajectory level. Specifically, the microbial abundance vector at the initial observation time $t_{0}$ was used as the model input, and the corresponding abundance trajectory at all observed time points was used as the supervision target. For dynamical model fitting, we used absolute abundance data directly. To remain consistent with the gLV formulation, microbial abundances were not log-transformed, converted to relative abundances, or rescaled before model fitting. The models were trained on absolute abundance trajectories. For NODE and gNODE, the loss function consists solely of the discrepancy between predicted and observed trajectories, as given in Equations 4, 6, respectively. For pNODE, the loss function includes both the data-fitting term and the gLV residual term defined in Equation 7, and the total weighted loss is given in Equation 8, with $w_{\text{data}}$ and $w_{\text{ODE}}$ each set to 0.5.

All models were trained using pre-specified hyperparameters. Specifically, NODE, pNODE, and gNODE were trained with the Adam optimizer, a learning rate of 0.001, a batch size equal to 20% of the number of subjects, and 500 training epochs. NODE and pNODE used the same two-layer MLP architecture for the ODE function, whereas gNODE used the gLV-parameterized ODE form. For trajectory prediction in the simulation studies, subject-level five-fold cross-validation was used, with 80% of the subjects used for training and the remaining 20% held out for testing in each fold. For the real datasets, leave-one-subject-out cross-validation was used to evaluate trajectory prediction performance. The held-out test subjects were used only for final performance evaluation.

Before training, the ecological parameters in gNODE and pNODE were initialized as follows: intrinsic growth rates $\alpha_{j}$ were sampled from a uniform distribution on (0,1), and off-diagonal interaction coefficients $\beta_{jk}$ were sampled from a normal distribution with mean 0 and standard deviation 0.1. The diagonal elements $\beta_{jj}$ were initialized at −1 to represent self-inhibition of each species. These biologically motivated initialization settings, including positive initialization of intrinsic growth rates and negative initialization of self-interaction terms, were used to promote ecologically plausible parameter estimates and improve numerical stability during model training.

### 2.6 Simulation study design

#### 2.6.1 Standard gLV simulation settings and perturbation settings

The simulation study was designed to systematically evaluate the performance of gNODE under controlled conditions, with a particular focus on interaction parameter estimation, trajectory prediction, and perturbation response prediction. These simulations were intended to reflect application scenarios involving sparse longitudinal sampling, small- to medium-sized microbial communities, and interpretable dynamical inference. To this end, we simulated microbial community dynamics from the gLV system under multiple scenarios (**Table 1**).

**Table 1:** Parameter settings for simulated data.

Parameter	Generation	Description
$y_{0}$	Uniform(0, 1)	Initial species abundance
$\alpha_{j}$	Uniform(0, 1)	Growth rate
$\beta_{jk}$	$j\neq k:(1-\pi )\cdot N(0,0.1^{2})+\pi \cdot \delta_{0}$ j=k: −1	Interaction matrix
π	{0, 0.2, 0.5, 0.8}	Sparsity parameter
S	{10, 20, 40, 60}	Number of subjects
P	{10, 20, 30}	Number of microbes
T	{3, 5, 10, 15, 20, 25}	Number of time points

For parameter settings, the initial abundance vector $y_{0}$ was sampled from a uniform distribution on (0,1). For off-diagonal interaction coefficients ( j≠k), $\beta_{jk}$ followed a mixture distribution: with probability 1−π, $\beta_{jk}$ was drawn from a normal distribution with mean 0 and standard deviation 0.1, and with probability π, it was set to 0. We considered four sparsity levels with π=0, 0.2, 0.5, and 0.8. For the diagonal terms ( j=k), we set $\beta_{jj}=-1$ to represent self-inhibition, and the growth rates $\alpha_{j}$ were independently sampled from a uniform distribution on (0,1).

Regarding community size, we considered communities with 10, 20, or 30 species, and 10, 20, 40, or 60 subjects. To mimic realistic sampling schemes, both the number and spacing of time points were varied: the number of time points was set to 3, 5, 10, 15, 20, or 25, and the time intervals were randomly drawn between 1 and 12 time units. For numerical integration, we used the ODE solver of the *deSolve* R package to solve the gLV system (Brown et al., 1989).

To assess perturbation responses, we conducted knock-out experiments for each species to examine the impact of species removal on community dynamics. Specifically, for a given species, we set its initial abundance to 0 and its associated interaction coefficients to 0, and then re-simulated the community dynamics under this knock-out configuration. In addition, we used the driver score metric to evaluate the importance of microbial species. This metric was derived from our previously proposed mbDriver framework (Tan et al., 2024). Specifically, the driver score is calculated based on the inferred ecological parameters and quantifies the impact of a given species on the overall dynamical state of the community by comparing the magnitude of changes in community steady states before and after the removal of that species, thereby assessing its importance within the microbial community.

#### 2.6.2 Stability analysis under repeated random initializations

To assess the stability of parameter estimation and downstream driver identification, we refitted gNODE 50 times with different random initializations across representative simulation scenarios. Parameter stability was summarized by sign consistency, empirical standard deviation, and empirical 95% interval width, while driver score stability was evaluated using pairwise Spearman correlations and Top-5 Jaccard indices across refits.

#### 2.6.3 Model misspecification analysis

To evaluate the robustness of gNODE under model misspecification, we additionally simulated microbial trajectories from a nonlinear saturating-response system rather than the standard gLV model:

(9) $$y_{ij}^{'}(t)=\alpha_{j}y_{ij}(t)+\sum_{k=1}^{P}\beta_{jk}y_{ij}(t)\frac{y_{ik}(t)}{H+y_{ik}(t)}$$

Here, H is the saturation constant controlling the degree of nonlinear saturation. We considered three saturation levels, H=0.1, 0.3 and 0.5. This analysis was conducted across representative simulation scenarios. Apart from replacing the standard gLV equation with the above saturating-response system, the parameter generation scheme, training procedures, and RMSE-based evaluation criteria were kept consistent with those used in the original trajectory prediction simulations.

### 2.7 Real data applications

Two experimental datasets were used to evaluate the performance of gNODE: a *Clostridioides difficile* infection dataset and a probiotic cocktail colonization dataset (Bucci et al., 2016). Both datasets were specifically chosen to test dynamic modeling performance under biologically relevant conditions. They provide longitudinal measurements that capture temporal changes in community composition, incorporate controlled perturbations to probe system responses, and report absolute bacterial abundances, which are essential for modeling intervention-associated biomass changes (Bucci and Xavier, 2014; Gerber, 2014). Together, these properties allow the datasets to reflect key aspects of microbial community dynamics, providing a robust basis for evaluating dynamical inference methods. Further details about these two datasets can be found in the Results section.

For the real-data analyses, we used the taxon-level longitudinal abundance profiles provided by the original study, rather than reprocessing raw sequencing reads from upstream bioinformatics pipelines. The real datasets were organized using the same data representation and input encoding strategy described in Section 2.5, with the initial abundance vector used as the model input and the corresponding longitudinal abundance trajectory used as the supervision target.

### 2.8 Bioinformatics and statistical analysis

In the simulation evaluation, to quantify the uncertainty in performance estimates and differences between methods, we calculated the mean relative RMSE and its 95% confidence interval across 10 independent data replications for each simulation setting. The 95% confidence interval was calculated using the t-distribution. In addition, we performed paired t-tests based on replication-level relative RMSEs to compare performance differences between methods under the same simulation setting, and adjusted the p-values for multiple testing using the Benjamini–Hochberg false discovery rate (FDR) method. The mean difference was defined as the first method minus the second method; therefore, a negative value indicates that the first method achieved a lower relative RMSE, and thus better performance.

For the real data analyses, the Shannon and Simpson indices were used as α-diversity metrics to characterize within-sample microbial diversity. For comparisons between two groups, differences in α-diversity were assessed using the Wilcoxon rank-sum test. For comparisons involving more than two groups, overall differences were first assessed using the Kruskal–Wallis test, followed by pairwise Wilcoxon rank-sum tests. P-values from pairwise comparisons were adjusted using the Benjamini–Hochberg FDR method. β-diversity was evaluated using Bray-Curtis distances and visualized using principal coordinates analysis (PCoA), and statistical significance was determined by permutational multivariate analysis of variance (PERMANOVA). All α- and β-diversity analyses were conducted using the *vegan* package in R (v2.6-4), and data visualizations were generated with the *ggplot2* package (v3.4.2).

## 3 Results

### 3.1 Overview of the gNODE framework

**Figure 1:**
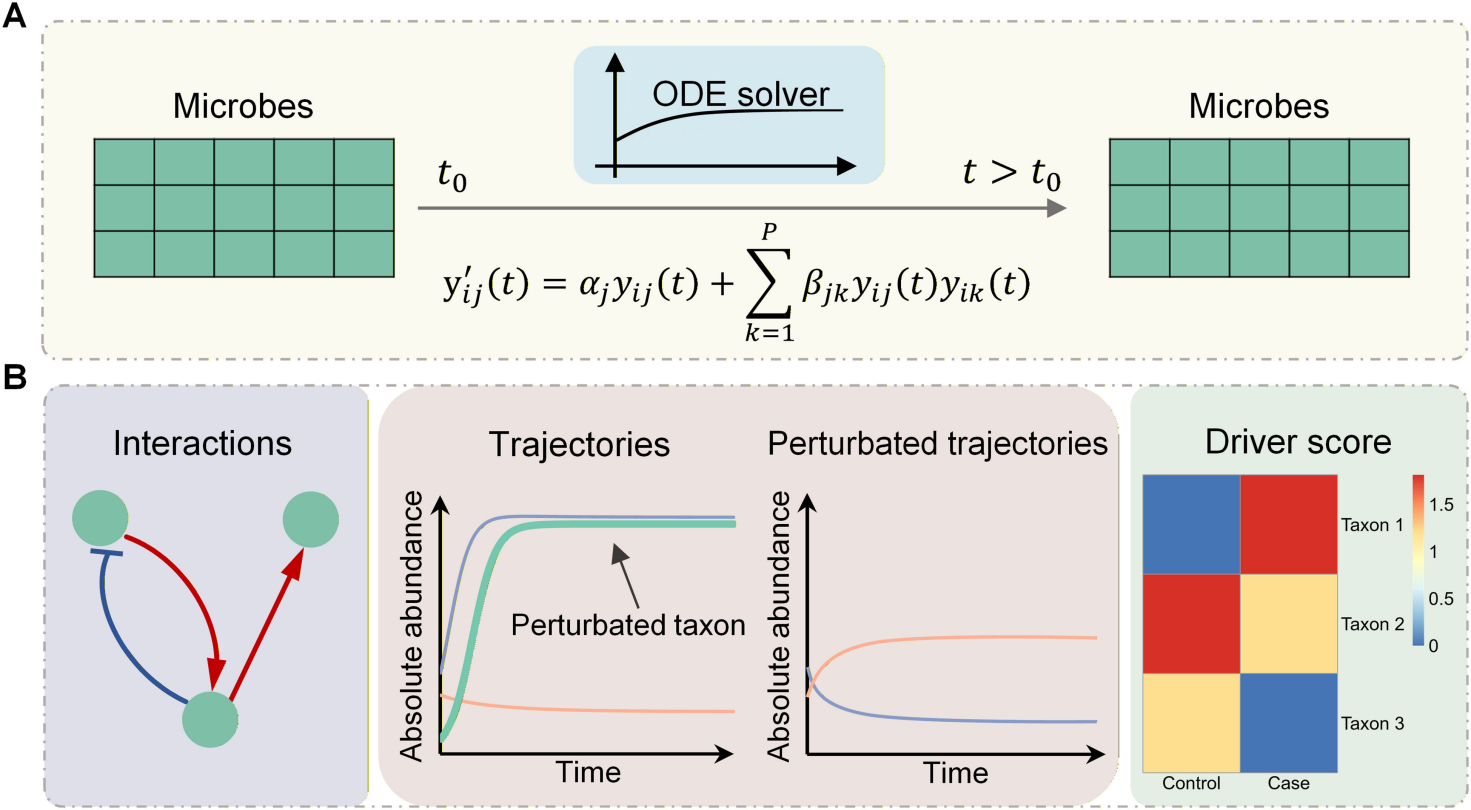
Schematic of the gNODE framework. **(A)** Training module: Longitudinal microbial abundance data are used to train a NeuralODE whose dynamics are defined by the gLV equations, enabling joint estimation of species-specific growth rates and interaction coefficients. **(B)** Prediction module: The trained model supports ecological network inference, forecasting of community trajectories, and simulation of system responses under perturbation scenarios. The inferred ecological parameters are further used to compute driver scores, which quantify each species’ contribution to community structure and dynamics.

The overall structure of gNODE is illustrated in **Figure 1**. As an ecology-guided deep learning framework, gNODE integrates ecological dynamical equations with NeuralODEs to enable parameter inference, community trajectory prediction, and perturbation response prediction from sparsely sampled microbiome time series. First, gNODE models microbial population dynamics using the gLV equations, which encode intrinsic growth rates and pairwise ecological interactions. These ecological equations are embedded directly into a NeuralODE architecture, allowing the model to learn species-specific growth and interaction parameters from longitudinal abundance data. Second, leveraging the inferred ecological network, gNODE predicts community trajectories at unobserved time points by solving the corresponding initial value problem with an ODE solver and simulates community responses under alternative perturbation scenarios, such as the removal or alteration of individual taxa. Finally, gNODE quantifies the functional importance of species by computing driver scores from the learned ecological parameters, thereby characterizing each taxon’s contribution to community structure and dynamic behavior. Taken together, these components enable gNODE to provide interpretable, dynamical, and perturbation-aware insights into microbiome community ecology.

### 3.2 Validation of gNODE using simulated datasets

We evaluated the performance of gNODE on simulated data in terms of interaction parameter estimation, trajectory prediction, and perturbation response prediction. To systematically assess robustness and applicability under different conditions, we varied the number of species, subjects, sampling time points, and the sparsity of the interaction matrix. gNODE was compared against several baseline methods, including a spline-based Ridge regression estimator (sRidge) (Tan et al., 2024), a standard NeuralODE model without gLV structural constraints (NODE), and a PINN-style variant (pNODE). These baseline methods were selected to represent the three methodological categories most directly related to this study: conventional two-step gLV parameter estimation, black-box NeuralODE modeling without ecological structural constraints, and physics-informed NeuralODE modeling with residual-based dynamical constraints. This comparison was designed to evaluate whether directly embedding gLV dynamics into the NeuralODE architecture improves interaction parameter estimation, trajectory prediction, and perturbation response modeling. Model performance was quantified using relative root mean squared error (RMSE), which measures discrepancies between predictions and ground truth.

**Figure 2:**
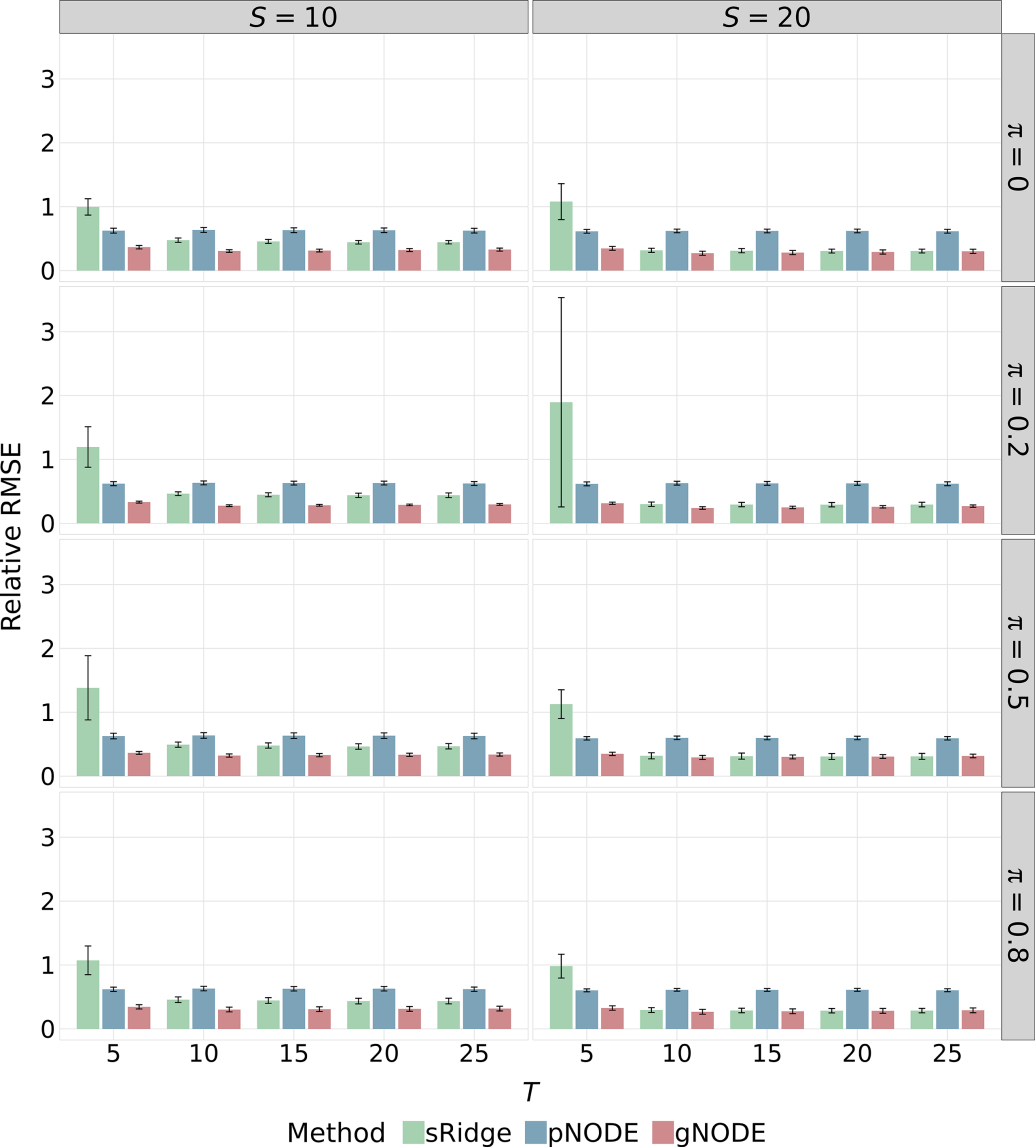
Performance of different methods in interaction parameter estimation. Simulated data were generated from the gLV model with P=10 microbes, sparsity parameter π∈{0, 0.2, 0.5, 0.8}, varying numbers of subjects S∈{10, 20} and time points T∈{5, 10, 15, 20, 25}. Bars represent the mean relative RMSE across 10 independent data replications, and error bars indicate 95% confidence intervals.

For interaction parameter estimation, we examined the performance of sRidge, pNODE, and gNODE in the case of P=10 species across different sparsity levels π∈{0, 0.2, 0.5, 0.8}, subject numbers S∈{10, 20}, and time points T∈{5, 10, 15, 20, 25}. Because NODE does not explicitly incorporate the gLV equations, its learned parameters lack direct ecological interpretability and were therefore excluded from the parameter estimation comparison. As shown in **Figure 2**, gNODE consistently achieved the lowest relative RMSE across all settings, indicating accurate and stable parameter estimation, with estimation error further decreasing as the number of time points increased. In contrast, sRidge performed poorly when the number of time points was small (particularly at T=5), likely because spline smoothing could not adequately capture temporal variation, leading to oversmoothing or underfitting. Its performance improved as more observations became available, suggesting that sRidge is better suited to longer time series. pNODE exhibited relatively large relative RMSE across scenarios, indicating limited utility for interaction parameter estimation. Overall, the sparsity π had only a modest effect on performance for all methods, and its impact on gNODE was especially small, suggesting that gNODE accommodates interaction networks across a wide range of sparsity levels. Error bars in **Figure 2** indicate 95% confidence intervals calculated across the independent data replications, and formal pairwise comparisons among sRidge, pNODE, and gNODE are provided in **Supplementary Table 1**.

**Figure 3:**
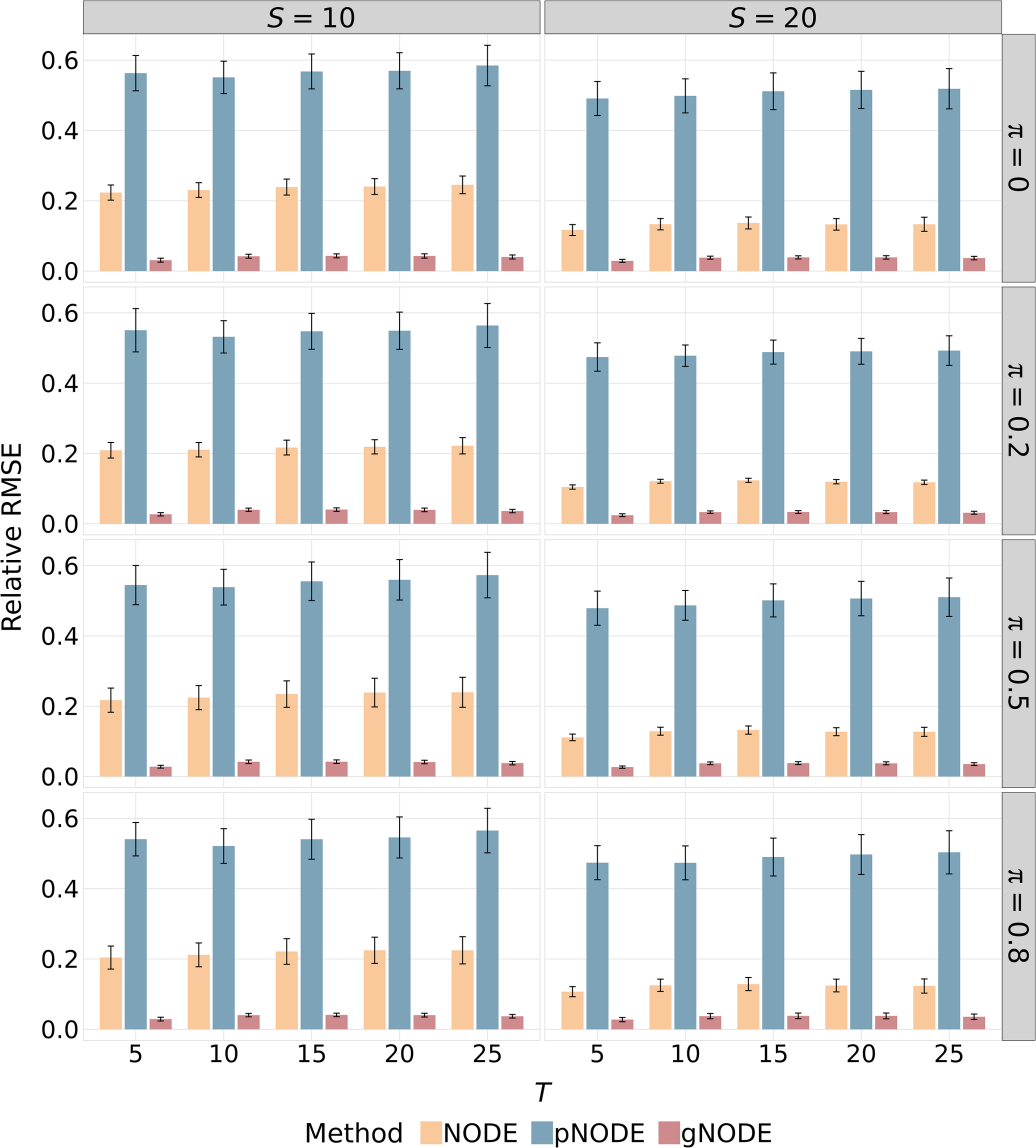
Performance of different methods in trajectory prediction. Simulated data were generated from the gLV model with P=10 microbes, sparsity parameter π∈{0, 0.2, 0.5, 0.8}, varying numbers of subjects S∈{10, 20} and time points T∈{5, 10, 15, 20, 25}. Bars represent the mean relative RMSE across 10 independent data replications, and error bars indicate 95% confidence intervals. Within each data replication, relative RMSE was first averaged across the five cross-validation folds.

To evaluate predictive performance on community trajectories, we used subject-level five-fold cross-validation, with 80% of subjects used for training and 20% held out as an independent test set in each fold. As shown in **Figure 3**, gNODE consistently achieved the lowest relative RMSE across most settings and maintained robust predictive performance under small sample sizes and sparse temporal sampling. By contrast, NODE generally outperformed pNODE in trajectory prediction, which may reflect increased optimization difficulty introduced by the PINN-style residual constraints in pNODE, potentially degrading predictive accuracy. For this analysis, relative RMSE was first averaged across the five cross-validation folds within each data replication, and error bars in **Figure 3** indicate 95% confidence intervals calculated across the independent data replications; formal pairwise comparisons among NODE, pNODE, and gNODE are provided in **Supplementary Table 2**. To further assess whether the models captured continuous dynamics rather than only minimizing aggregate RMSE, we visualized representative held-out trajectories. As shown in **Supplementary Figures 2**–**6**, gNODE generally produced trajectories that better matched the held-out observations and temporal patterns across different sampling densities, whereas NODE and especially pNODE showed larger deviations for several species. These visual results are consistent with the cross-validation RMSE results.

**Figure 4:**
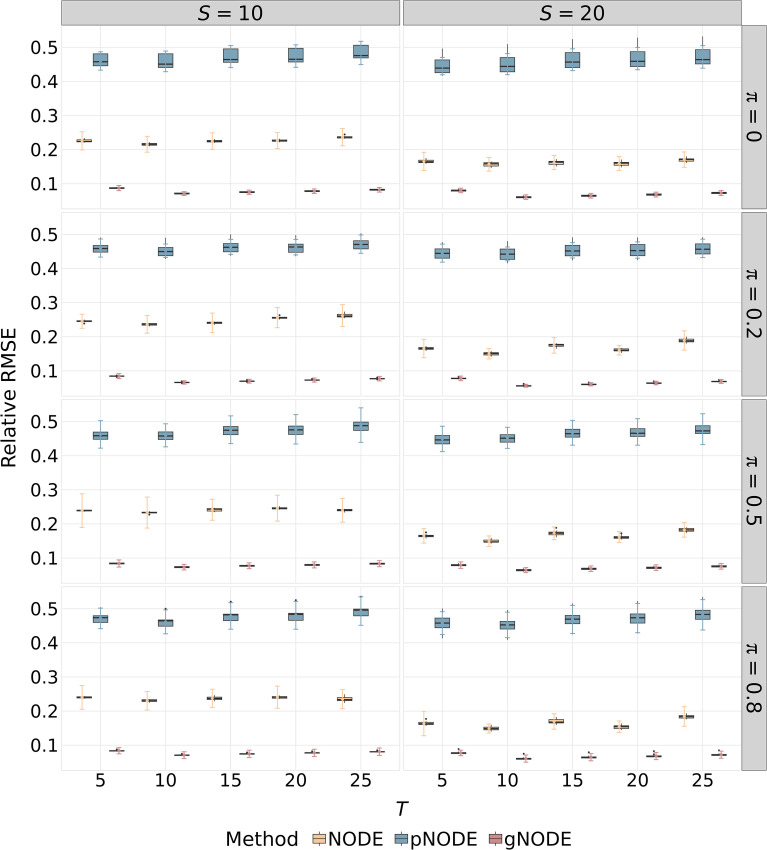
Performance of different methods in perturbation response prediction. Simulated data were generated from the gLV model with P=10 microbes, sparsity parameter π∈{0, 0.2, 0.5, 0.8}, varying numbers of subjects S∈{10, 20}, and time points T∈{5, 10, 15, 20, 25}. Boxplots represent the distribution of subject-level relative RMSEs. Overlaid points represent the mean relative RMSE across 10 independent data replications, and error bars indicate 95% confidence intervals.

We next evaluated perturbation response prediction using single species knock-out experiments to assess generalization under local perturbations. For each scenario, predicted trajectories under species removal were compared with the corresponding simulated ground truth. **Figure 4** shows the distribution of subject-level relative RMSEs, and formal pairwise comparisons among NODE, pNODE, and gNODE are provided in **Supplementary Table 3**. These results show that gNODE consistently yielded the lowest prediction error across all conditions, highlighting its advantage in capturing community responses to perturbations.

We further compared gNODE with other methods under more challenging simulation settings, including larger numbers of species and fewer sampling time points. Across these additional scenarios, gNODE consistently outperformed competing approaches in interaction parameter estimation, trajectory forecasting, and perturbation response prediction (**Supplementary Figures 7**–**9**). Together with the main simulation results, these findings indicate that, for data generated from gLV-based ecological dynamical models, gNODE provides accurate and robust estimation of the ecological network, reliable trajectory prediction, and improved characterization of perturbation responses. These results also provide practical guidance on the required temporal sampling density. Under the simulation settings considered here, gNODE could be trained with as few as 3 time points and remained relatively stable. However, the required number of time points is not a universal fixed threshold and depends on the modeling objective, community size, sample size, noise level, and temporal complexity of the system. In practice, more time points are recommended for accurate interaction parameter estimation and perturbation response prediction whenever experimentally feasible.

Given the potential identifiability concerns of gLV interaction parameters, we further evaluated the stability of the inferred interaction parameters and downstream driver scores by repeatedly fitting gNODE with different random initializations. Under the setting P=10, S=10, π=0.5, and T∈{5,10,15,20,25}, gNODE was refitted 50 times for each time-point setting. The inferred interaction coefficients showed moderate directional stability, with an average sign consistency of approximately 0.75 across all coefficients and 0.72–0.74 for off-diagonal coefficients (**Supplementary Figure 10**). The empirical standard deviation and empirical 95% interval width of off-diagonal interaction coefficients were largest at T=5, decreased substantially at T=10, and remained relatively stable thereafter (**Supplementary Table 4**). These results suggest that the inferred interaction structure has some directional robustness, although the precise numerical values of individual coefficients remain uncertain. We further assessed the stability of downstream driver scores. Across repeated refits, the average pairwise Spearman correlation of driver scores was approximately 0.90, and the average pairwise Jaccard index of Top-5 driver sets ranged from 0.77 to 0.84 (**Supplementary Figure 11**). These results indicate that, although individual gLV parameters may vary across repeated fits, the overall driver ranking and key driver identification are more stable.

Finally, to evaluate robustness under model misspecification, we employed a nonlinear saturating-response system. As this misspecified system was not generated from a standard gLV model, there was no directly comparable ground-truth gLV interaction matrix available; thus, this analysis focused on trajectory prediction performance. As shown in **Supplementary Figure 12**, gNODE maintained stable and competitive predictive performance under this misspecified setting, particularly when H=0.1. Although NODE achieved lower prediction errors in some settings, gNODE consistently outperformed pNODE and remained relatively stable across different temporal sampling densities. These results suggest that gNODE’s predictive performance is not solely due to the correctly specified gLV simulation design. They also indicate that the embedded gLV structural constraint, while enhancing interpretability and stability, may reduce predictive flexibility when true dynamics deviate significantly from the standard gLV form.

### 3.3 Application to real datasets

#### 3.3.1 Analysis of the *Clostridioides difficile* infection dataset

This dataset originates from a longitudinal study of *Clostridioides difficile* infection (CDI) (Bucci et al., 2016). *C. difficile* can cause intestinal infections, leading to diarrhea and other gastrointestinal disorders. CDI is frequently associated with antibiotic use, as antibiotics disrupt the gut microbiota and create ecological niches that facilitate *C. difficile* overgrowth and toxin production. Although probiotic supplementation can reduce infection risk, the specific gut microbes that confer colonization resistance against *C. difficile* and the mechanisms underlying this protection remain incompletely understood (Buffie et al., 2015).

We applied gNODE to the CDI dataset to predict microbial taxa with inhibitory potential against *C. difficile* growth. The dataset includes five mice followed over a 56-day experimental period, with *C. difficile* introduced on day 28. Fecal samples were collected at 13 time points before infection (Control group) and 13 time points after infection (Case group), yielding 26 samples per mouse. Microbial abundances were profiled by high-throughput 16S rRNA sequencing, and total bacterial biomass was quantified by 16S rRNA qPCR using universal primers. After taxonomic annotation, 16 species were identified across 130 samples. To characterize community-level differences between the pre- and post-infection phases, we calculated α-diversity using the Shannon and Simpson indices. As shown in **Supplementary Figures 13A, B**, both richness and evenness were significantly different between the Case and Control groups (P < 0.05). We then assessed β-diversity using Bray-Curtis distances and visualized differences in community composition with PCoA. As shown in **Supplementary Figure 13C**, the microbial communities of the two phases differed significantly (PERMANOVA, P = 0.001). Together, these analyses indicate a pronounced community shift following infection.

**Figure 5:**
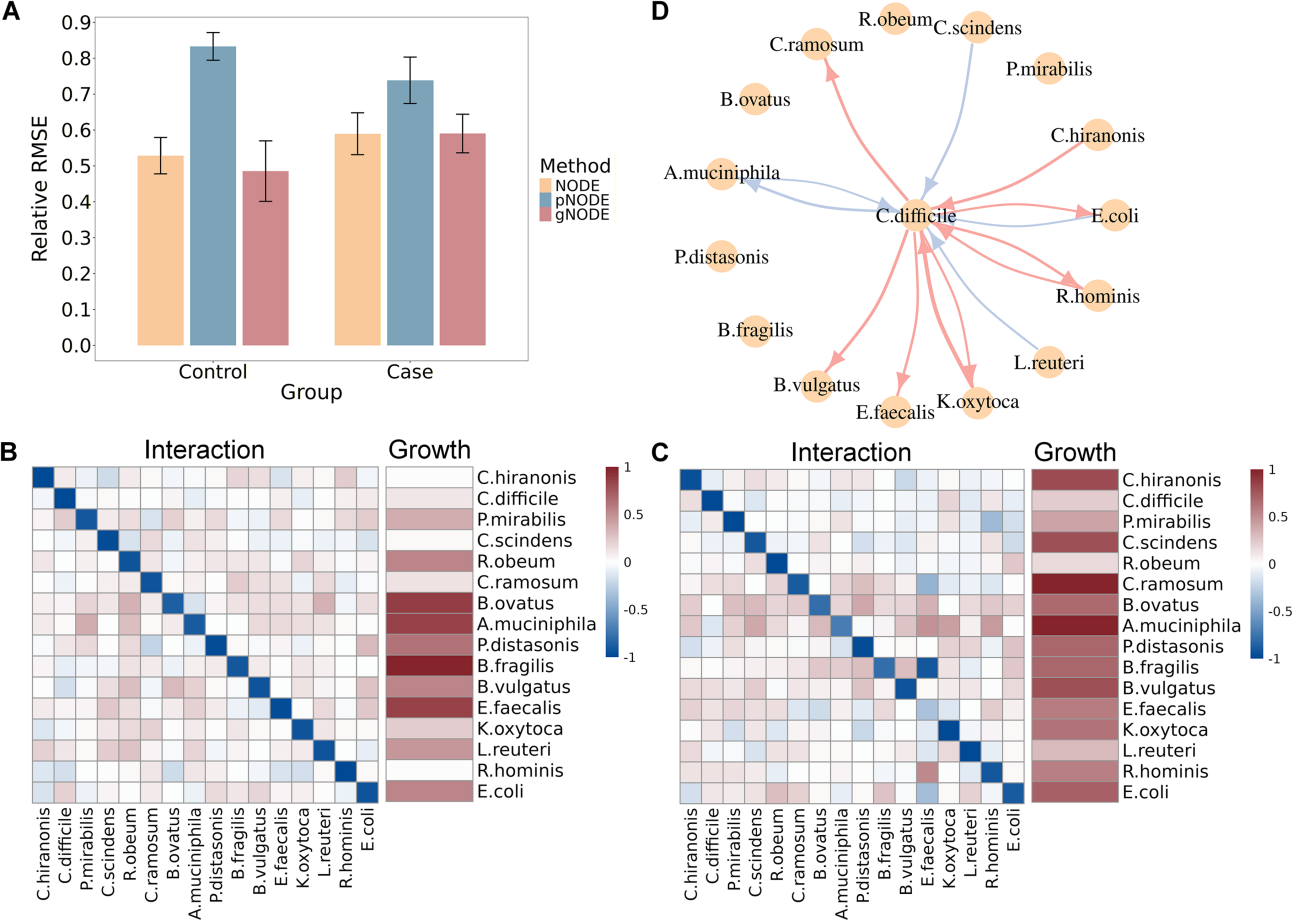
gNODE inference of microbial dynamics and interactions in the *C. difficile* infection dataset. **(A)** Comparison of trajectory prediction performance across methods in the *C. difficile* infection dataset. **(B, C)** Microbial intrinsic growth rates and interactions inferred by gNODE before **(B)** and after **(C)** *C. difficile* intervention. **(D)** Interaction network between *C. difficile* and other microbial species. Each node represents a species; red arrows denote positive (activating) interactions, and blue arrows denote negative (inhibitory) interactions. Arrow thickness reflects interaction strength, with thicker arrows indicating stronger effects.

We evaluated the predictive performance of gNODE using leave-one-out cross-validation at the individual level (i.e., leaving one mouse out for testing) and compared it with pNODE and NODE. gNODE achieved superior predictive accuracy in both Control and Case groups, underscoring the benefit of incorporating ecological dynamical equations (**Figure 5A**). After establishing strong predictive performance in trajectory prediction, we next applied gNODE to infer microbial interaction networks from the full dataset. As shown in **Figures 5B, C**, the inferred parameters adhered to biologically plausible constraints, including nonnegative intrinsic growth rates and negative self-interaction terms. The off-diagonal coefficients captured both positive and negative species interactions, revealing shifts in microbial relationships between the pre- and post-infection phases.

We further examined the inferred post-infection interaction network. As shown in **Figure 5D**, predicted inhibitory taxa included *Clostridium scindens*, *Lactobacillus reuteri*, *Akkermansia muciniphila*, and *Escherichia coli*, among which several have supporting evidence for antagonism against *C. difficile*. *C. scindens* possesses 7α-dehydroxylation activity, enabling the conversion of primary bile acids into secondary bile acids such as deoxycholic acid, which has been shown to inhibit *C. difficile* growth (Usui et al., 2020). Its inhibitory role has also been validated in mouse models through its modulation of host bile acid metabolism (Buffie et al., 2015). In addition, *L. reuteri* is a probiotic bacterium capable of producing reuterin, a broad-spectrum antimicrobial compound that suppresses *C. difficile* growth by generating reactive oxygen species and disrupting its metabolism (Engevik et al., 2020). *A. muciniphila* has been reported to reduce *C. difficile* infection and associated intestinal inflammation by enhancing gut barrier integrity and modulating the gut microbiota and metabolome (Wu et al., 2022). Collectively, these results support the ability of gNODE to recover biologically meaningful microbial interactions from longitudinal data.

#### 3.3.2 Analysis of the probiotic cocktail colonization dataset

This dataset was designed to investigate the colonization dynamics of a probiotic cocktail in germ-free mice and its response to dietary fiber perturbation (Bucci et al., 2016). The probiotic cocktail has been shown to induce regulatory T cells and suppress inflammation, indicating its therapeutic potential for inflammatory bowel disease (IBD) (Atarashi et al., 2013). Given that diet is a major modulator of IBD through its effects on epithelial and immune cells and the gut microbiota (Gill et al., 2022), probiotic consortia intended for IBD therapy must remain stable and functionally effective under varying dietary conditions. Accordingly, this analysis aims to assess how a low-fiber diet alters community structure and to identify key strains driving these changes.

The dataset includes 13 Clostridia probiotic strains colonizing seven mice. Over the nine-week experiment, 56 fecal samples per mouse were collected, corresponding to near-daily sampling with occasional exceptions due to logistical constraints. Mice were fed a high-fiber diet for the first five weeks (High_fiber1 group), followed by a low-fiber diet for two weeks (Low_fiber group), and finally a restored high-fiber diet for the final two weeks (High_fiber2 group). To evaluate the global impact of dietary fiber perturbation on community composition, we first assessed α- and β-diversity across dietary phases. α-diversity, measured by the Shannon and Simpson indices, was significantly reduced in the Low_fiber group compared with both high-fiber phases (P < 0.05; **Supplementary Figures 14A, B**). Consistently, β-diversity analysis based on Bray-Curtis distances and PCoA showed that the two high-fiber phases were highly similar, whereas the Low_fiber group differed significantly from both (PERMANOVA, P = 0.001; **Supplementary Figure 14C**).

**Figure 6:**
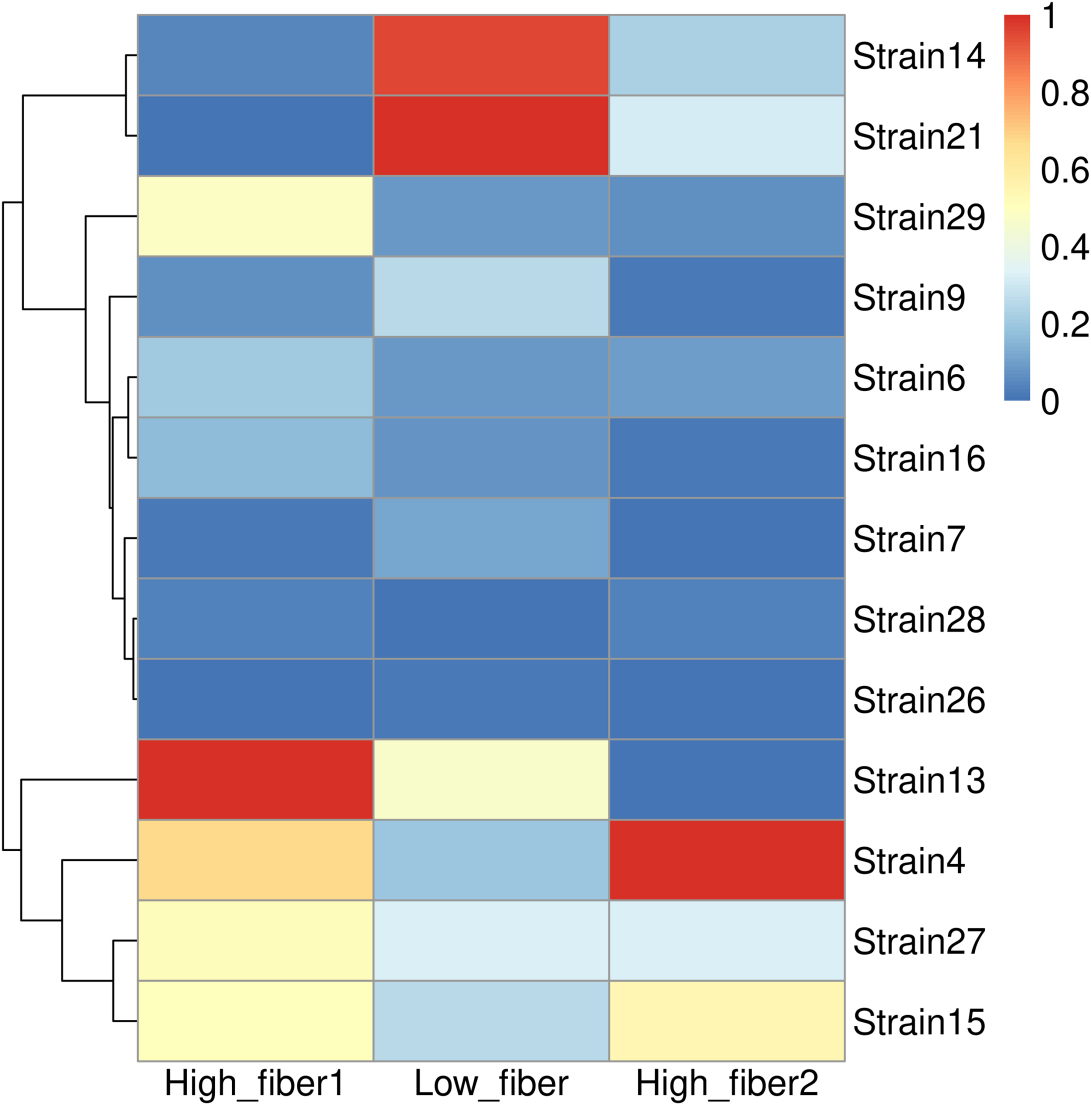
Predicted microbial driver scores in the probiotic cocktail dataset. The heatmap of predicted driver microbes across different dietary fiber intervention groups, with higher values indicating higher microbial driver scores.

Having established that dietary fiber deprivation induces a marked shift in community composition, we next applied gNODE to quantify the contribution of individual strains to community structure under different dietary conditions using the driver score. The driver score evaluates the importance of a strain by measuring the change in the community steady state upon its removal (Tan et al., 2024). As shown in **Figure 6**, strains 15, 4, and 27 exhibited consistently high driver scores in both high-fiber phases, indicating their central roles in maintaining community stability under fiber-rich conditions. Notably, previous studies have shown that strain 15 is most closely related to *Clostridium asparagiforme* (Bucci et al., 2016), a short-chain fatty acid-producing species that ferments dietary fiber and supports gut homeostasis. In contrast, under low-fiber conditions, strains 14 and 21 displayed the highest driver scores, suggesting a shift in dominant contributors to community structure in response to dietary perturbation. Strain 14 is closely related to *Ruminococcus* sp. *ID8* (Bucci et al., 2016), which may play a key role under low-fiber conditions.

To compare driver-based importance with conventional network connectivity metrics, we further calculated the degree centrality of each strain under different dietary conditions. Degree centrality reflects the number of connections associated with each strain in the inferred interaction network and serves as a measure of local network connectivity. As summarized in **Supplementary Table 5**, although strain 15 showed high degree centrality under high-fiber conditions, strains 4 and 27 did not rank among the most highly connected strains. Similarly, under low-fiber conditions, strains 14 and 21 did not exhibit the highest degree values. These findings indicate that strains exerting strong influence on community steady states are not necessarily those with the highest network connectivity, underscoring the limitations of purely connectivity-based metrics in capturing key contributors to community structure.

## 4 Discussion

Understanding microbial interactions is central to interpreting gut community dynamics. However, existing frameworks, including gLV models and deep learning approaches, are often constrained by high dimensionality, sparse temporal sampling, thereby limiting reliable parameter estimation and mechanistic interpretation. To address these challenges, we developed gNODE, a framework that integrates the gLV ecological model with NeuralODEs to enable both parameter inference and dynamic prediction. By embedding ecological equations into a deep learning architecture, gNODE adopts a physics-informed learning paradigm that supports robust parameter estimation even under sparse temporal sampling. Beyond parameter estimation, gNODE can forecast community trajectories and predict responses to external perturbations. Accordingly, gNODE is particularly suited for interpretable dynamical analysis of low- to moderate-complexity microbial communities with longitudinal absolute abundance measurements under defined experimental conditions, such as identifying condition-specific driver taxa or comparing community dynamics across different intervention stages.

We used simulated datasets to demonstrate that gNODE outperforms several competing approaches in interaction parameter estimation, trajectory forecasting, and perturbation response prediction. We further applied gNODE to two real datasets and showed that it effectively reconstructs biologically meaningful interaction networks. In the *C. difficile* infection dataset, gNODE inferred interaction matrices and growth parameters that adhered to known biological constraints and revealed inhibitory effects of multiple taxa, including *C. scindens*, *L. reuteri*, and *A. muciniphila*, on *C. difficile* growth (Engevik et al., 2020; Usui et al., 2020). In the probiotic cocktail dataset, gNODE identified strain-specific contributions under different dietary fiber conditions, highlighting its potential for assessing perturbation responses and guiding the design of probiotic consortia. We further emphasize that gNODE is intended as a reusable ecological dynamical modeling framework, rather than a universal pre-trained model with fixed parameters transferable across arbitrary datasets. Because gLV parameters are community- and condition-specific, applying gNODE to a new dataset generally requires re-estimating the dynamical parameters to obtain interpretable, condition-specific trajectory fitting, interaction inference, and perturbation response analysis.

However, several issues warrant further consideration and future extension. First, although the standard gLV formulation improves mechanistic interpretability, explicitly embedding the gLV model within the NeuralODE framework may introduce model misspecification when the true microbial dynamics deviate substantially from the standard gLV form. Our saturating-response simulations showed that gNODE retained stable predictive performance under a representative nonlinear misspecification scenario; however, this analysis does not fully cover context-dependent interactions or broader non-pairwise effects, such as higher-order interactions. Future extensions could allow growth rates and interaction coefficients to depend on environmental and host-related covariates, and could explicitly incorporate higher-order terms to capture non-pairwise ecological effects. In addition, extending gNODE to stochastic differential equation or state-space model frameworks may improve robustness by explicitly modeling process noise and observation error in noisy real-world microbiome time-series data. Second, like other ecological dynamical models, the gLV formulation faces parameter identifiability issues, especially as community size increases. Different parameter combinations may generate highly similar trajectories, thereby increasing the difficulty of reliably inferring true ecological interactions (Angulo et al., 2017). To reduce the dimensionality of the parameter space and improve estimation stability under sparse longitudinal sampling, gNODE assumes that, within each fitted condition, different individuals share the same set of time-invariant dynamical parameters. Therefore, the inferred interaction matrix should be interpreted as a condition-specific, time-invariant average interaction structure shared across individuals within the fitted condition, rather than as an individual-specific or time-varying dynamic network. Potential directions to mitigate this challenge include jointly analyzing time-series and cross-sectional data, incorporating prior ecological knowledge, or, when denser longitudinal sampling is available, allowing growth rates and interaction coefficients to vary across individuals or over time. Third, our benchmark comparison focused on methods most directly related to the proposed framework and did not cover all ecological dynamical inference tools. Future work will include additional inference frameworks and more complex benchmark scenarios to comprehensively evaluate the scope of applicability and relative performance of gNODE. Finally, the current gNODE framework relies primarily on species abundance dynamics, whereas a more comprehensive understanding of microbial community function often requires the integration of additional omics data (Heintz-Buschart et al., 2016). Because gut metabolite profiles may outperform species-level abundances in predicting host health outcomes (Franzosa et al., 2018; Goyal et al., 2021), extending gNODE to incorporate microbe-metabolite interactions may enhance biological interpretability and broaden its applicability.

In summary, this study provides a new framework for predicting microbial community dynamics. However, gNODE was evaluated primarily using gnotobiotic animal datasets, whose simplified community structures favor interpretability and controlled benchmarking (Bucci et al., 2016). In contrast, microbiomes of conventionally raised animals and humans are substantially more complex and subject to diverse perturbations, presenting additional analytical challenges. Future work will focus on extending gNODE to more complex ecosystems and improving its scalability and performance in increasingly diverse and high-complexity microbial systems.

## Data Availability

Publicly available datasets were analyzed in this study. We applied gNODE to two real datasets. The Clostridioides difficile infection dataset and the probiotic cocktail colonization dataset were obtained from publicly available data (Bucci et al., 2016). The data and source code used in this study are deposited in the GitHub repository, available at: https://github.com/tanxiaoxiu/gNODE.
