# Supplementary material for: gNODE: gLV model-informed neural ordinary differential equations for modeling microbial community dynamics
Source: Front Cell Infect Microbiol. 2026 Jul 6;16:1785750. doi: 10.3389/fcimb.2026.1785750 (PMC13381248; doi:10.3389/fcimb.2026.1785750)
Supplement: Supplementary file 1 [file DataSheet1.pdf]

Supplementary Information

Supplementary Figures

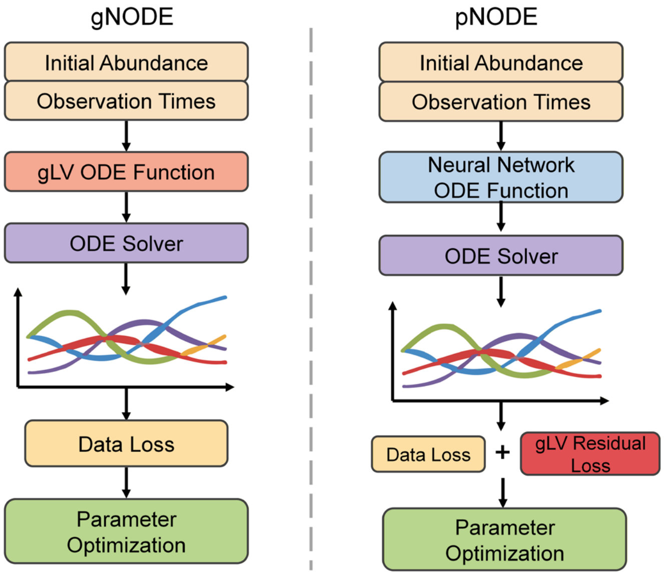

Supplementary Fig. 1 Schematic diagram of the implementation structures of gNODE and pNODE. gNODE directly embeds the gLV equations into the dynamical function of the NeuralODE, whereas pNODE adopts a standard NeuralODE framework and imposes ecological dynamical constraints through an additional gLV residual penalty.

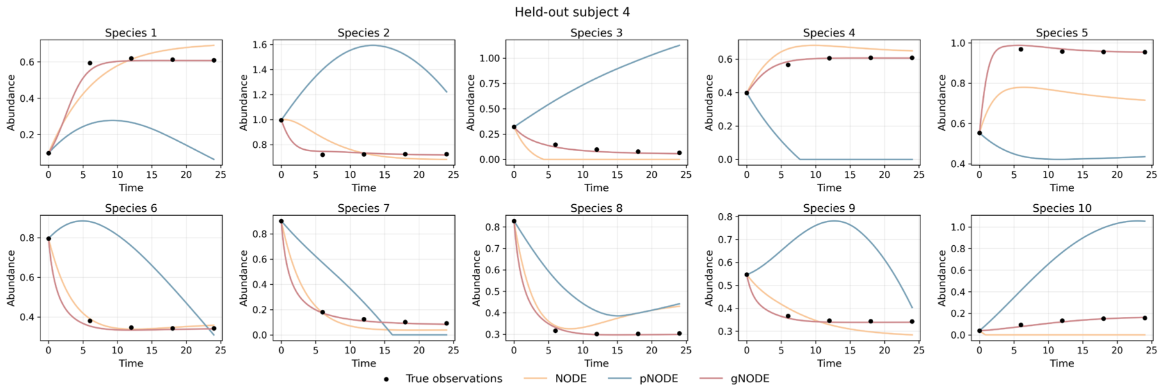

Supplementary Fig. 2 Representative trajectory prediction results of NODE, pNODE, and gNODE on a held-out test sample under the simulation setting with sparsity $\pi = 0.5$, $P = 10$, $S = 10$, data

replication $= 1$, and $T = 5$ time points. The figure shows the first held-out test subject in fold 1 of the five-fold cross-validation, corresponding to subject 4. Each panel represents one species (Species 1–10); black points denote the true observations, and the curves denote the continuous trajectories predicted by NODE, pNODE, and gNODE.

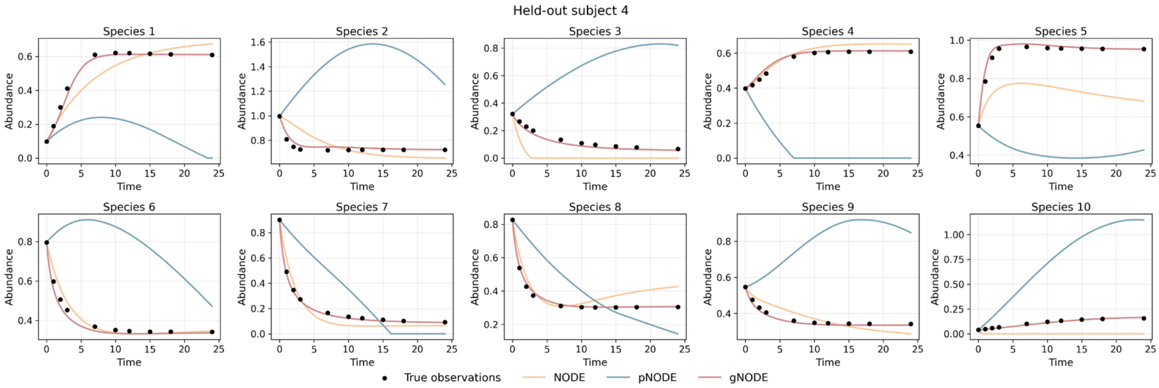

Supplementary Fig. 3 Same as Supplementary Fig. 2, but with $T = 10$.

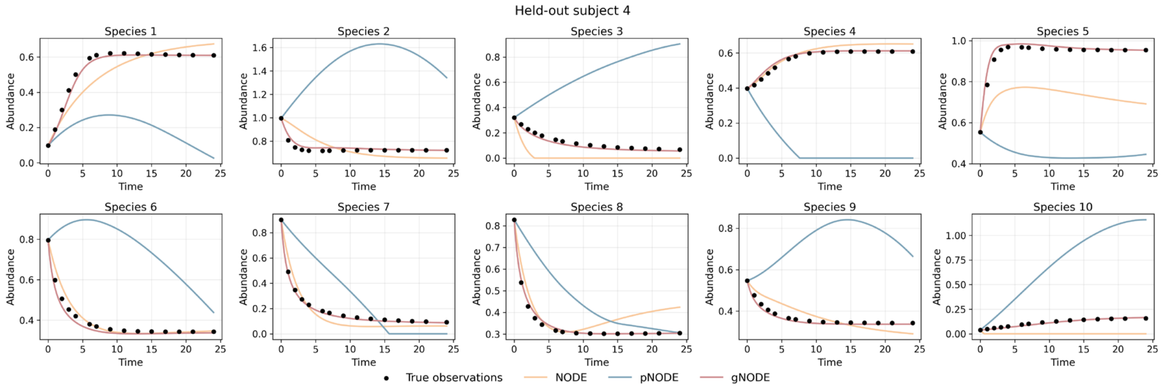

Supplementary Fig. 4 Same as Supplementary Fig. 2, but with $T = 15$.

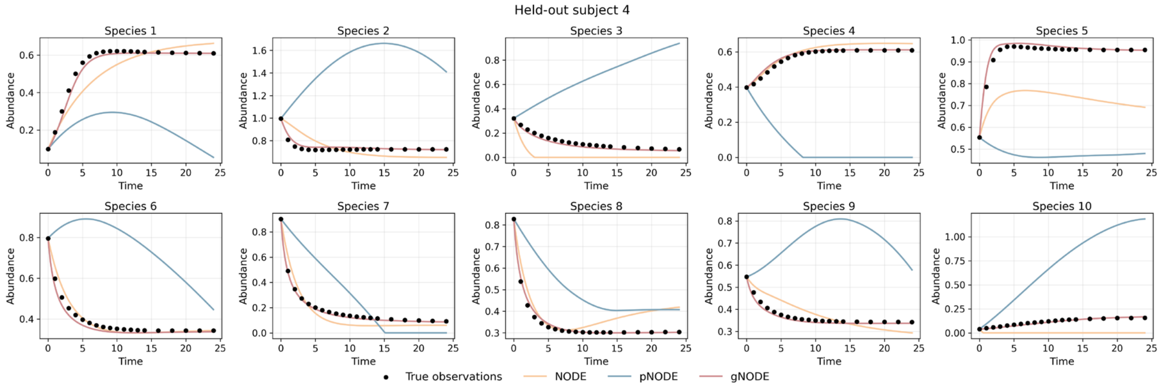

Supplementary Fig. 5 Same as Supplementary Fig. 2, but with $T = 20$.

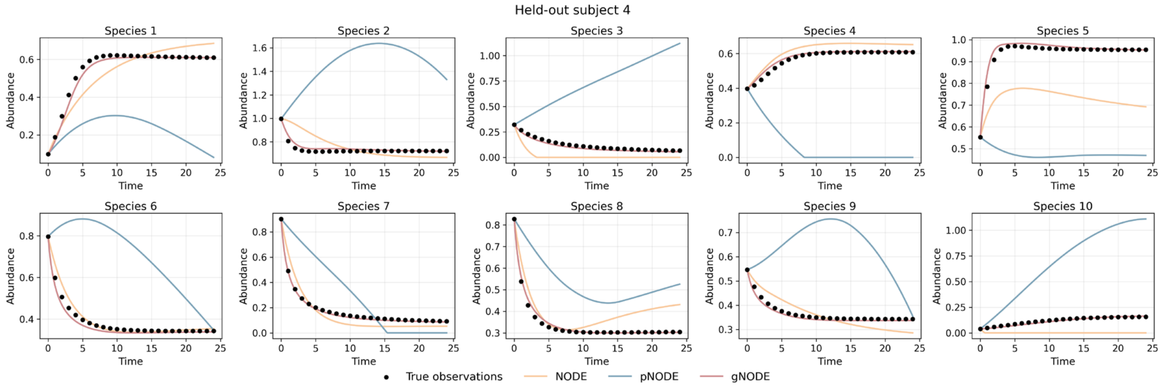

Supplementary Fig. 6 Same as Supplementary Fig. 2, but with $T = 25$.

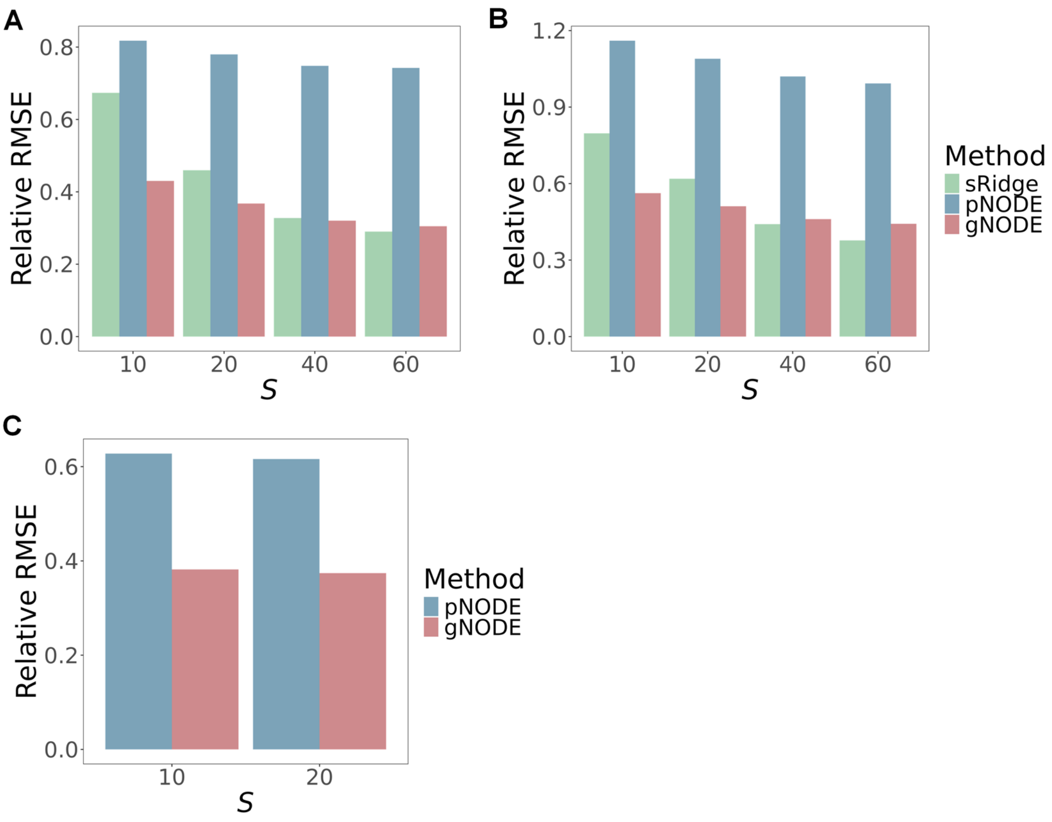

Supplementary Fig. 7 Performance of different methods in parameter estimation. Simulated data generated from the gLV model with a sparsity parameter $\pi = 0.2$ were used to evaluate the estimation performance of different methods under varying numbers of microbes, subjects, and time points. **(A)** Number of microbes $P = 20$, number of subjects $S \in \{10, 20, 40, 60\}$, and number of time points $T = 10$. **(B)** Number of microbes $P = 30$, number of subjects $S \in \{10, 20, 40, 60\}$, and number of time points $T = 10$. **(C)** Number of microbes $P = 10$, number of subjects $S \in \{10, 20\}$, and number of time points $T = 3$. The RMSE was averaged over 10 data replications.

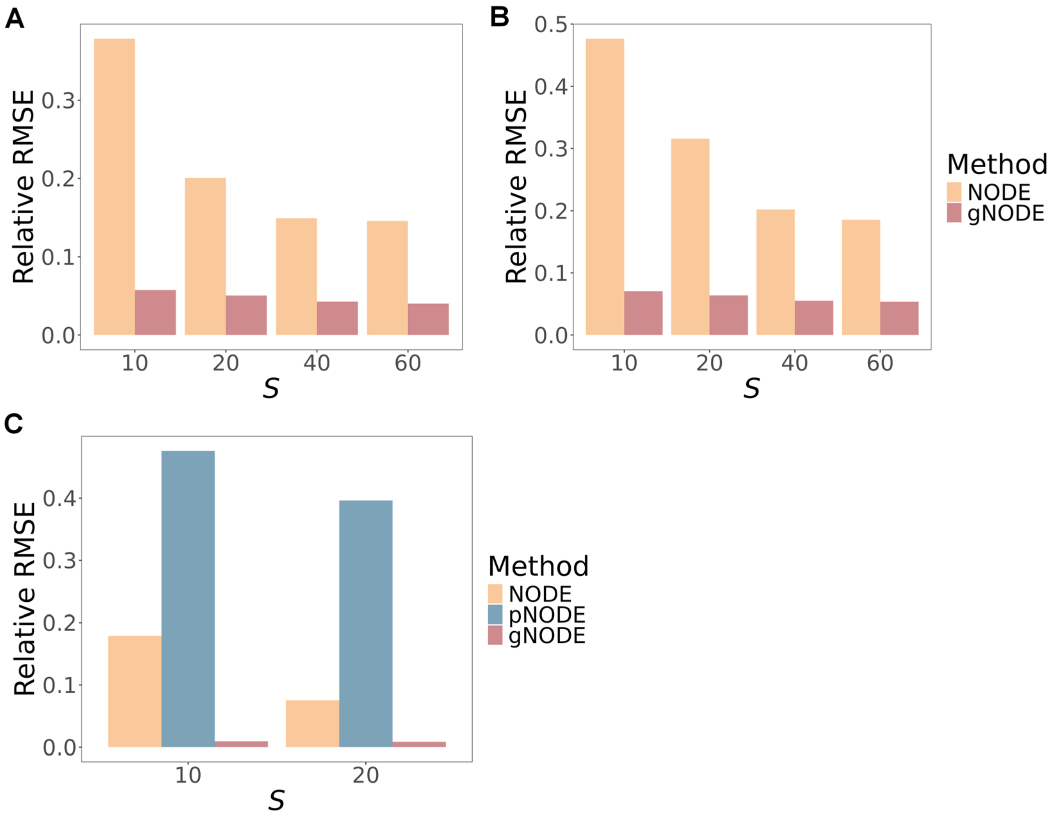

Supplementary Fig. 8 Performance of different methods in trajectory prediction. Simulated data generated from the gLV with a sparsity parameter $\pi = 0.2$ were used to evaluate the prediction performance of different methods under varying numbers of microbes, subjects, and time points. **(A)** Number of microbes $P = 20$, number of subjects $S \in \{10, 20, 40, 60\}$, and number of time points $T = 10$. **(B)** Number of microbes $P = 30$, number of subjects $S \in \{10, 20, 40, 60\}$, and number of time points $T = 10$. **(C)** Number of microbes $P = 10$, number of subjects $S \in \{10, 20\}$, and number of time points $T = 3$. The RMSE was averaged over 10 data replications.

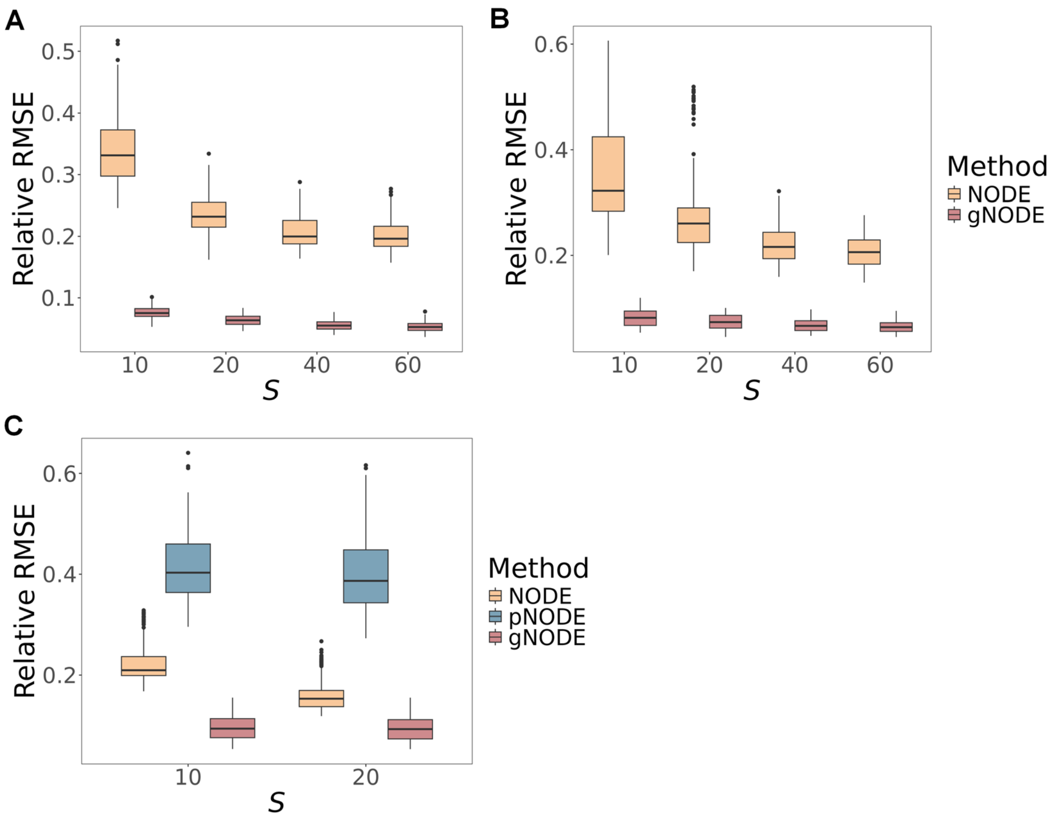

Supplementary Fig. 9 Performance of different methods in predicting intervention trajectories. Simulated data generated from the gLV with a sparsity parameter $\pi = 0.2$ were used to evaluate the prediction performance of different methods under varying numbers of microbes, subjects, and time points. **(A)** Number of microbes $P = 20$, number of subjects $S \in \{10, 20, 40, 60\}$, and number of time points $T = 10$. **(B)** Number of microbes $P = 30$, number of subjects $S \in \{10, 20, 40, 60\}$, and number of time points $T = 10$. **(C)** Number of microbes $P = 10$, number of subjects $S \in \{10, 20\}$, and number of time points $T = 3$. The RMSE was averaged over 10 data replications.

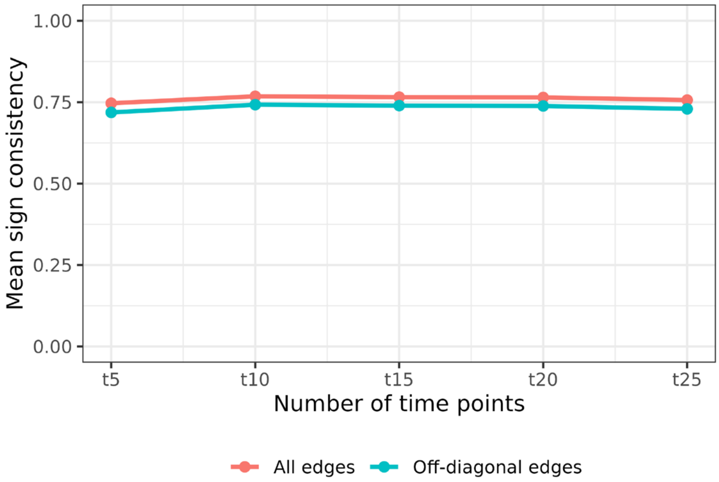

Supplementary Fig. 10 Sign consistency of inferred interaction parameters across different numbers of time points. For each time-point setting, gNODE was refitted 50 times using different random initializations. Mean sign consistency was computed across repeated refits. Results are shown separately for all interaction coefficients and for off-diagonal coefficients only.

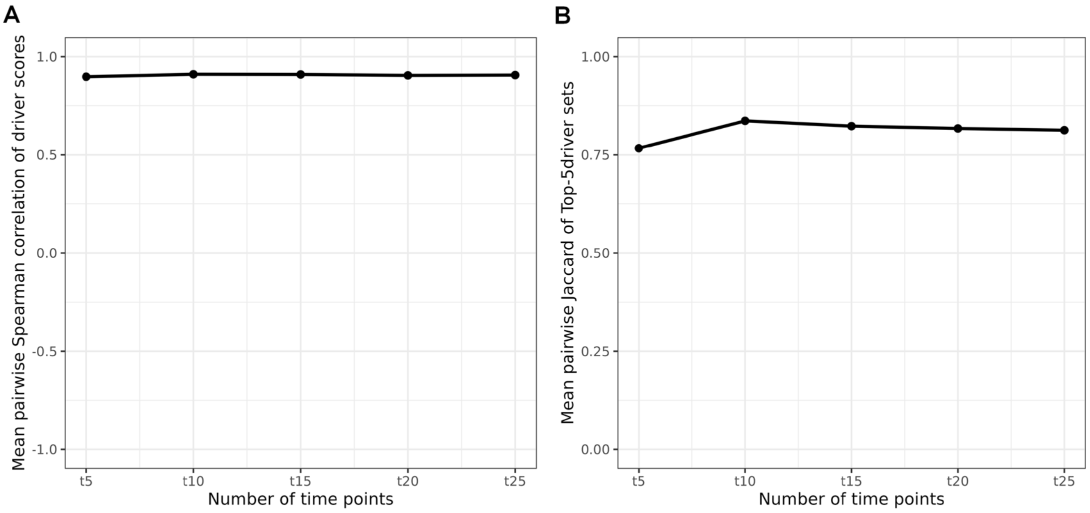

Supplementary Fig. 11 Stability of downstream driver identification across different numbers of time points. For each time-point setting, gNODE was refitted 50 times using different random initializations. **(A)** Mean pairwise Spearman correlation of driver scores across refits. **(B)** Mean pairwise Jaccard index of Top-5 driver sets across refits.

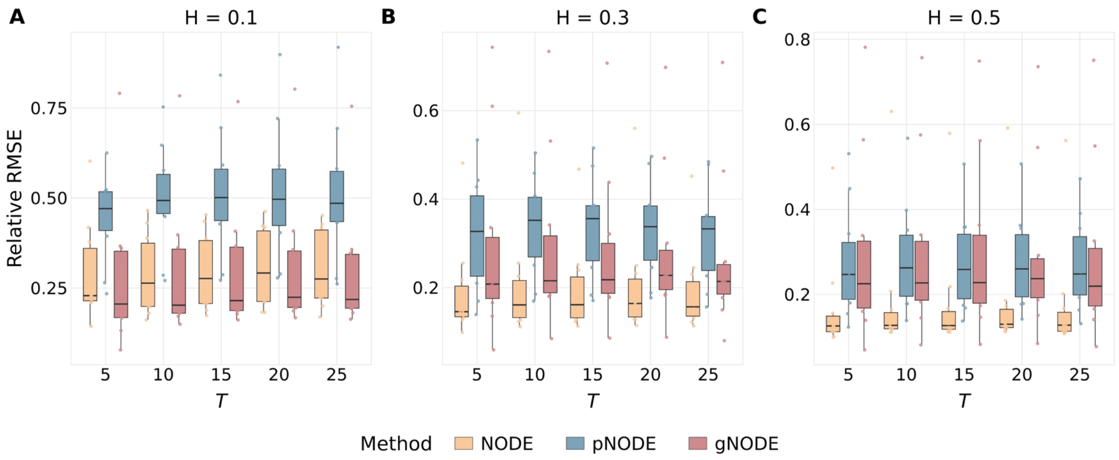

Supplementary Fig. 12 Performance of different methods in trajectory prediction under model misspecification. Simulated data were generated from the nonlinear saturating-response system with a sparsity parameter $\pi = 0.5$, number of microbes $P = 10$, and number of subjects $S = 10$. The prediction performance of NODE, pNODE, and gNODE was evaluated under varying numbers of time points $T \in \{5,10,15,20,25\}$. **(A)** Saturation constant $H = 0.1$. **(B)** Saturation constant $H = 0.3$. **(C)** Saturation constant $H = 0.5$. The RMSE was averaged over 10 data replications.

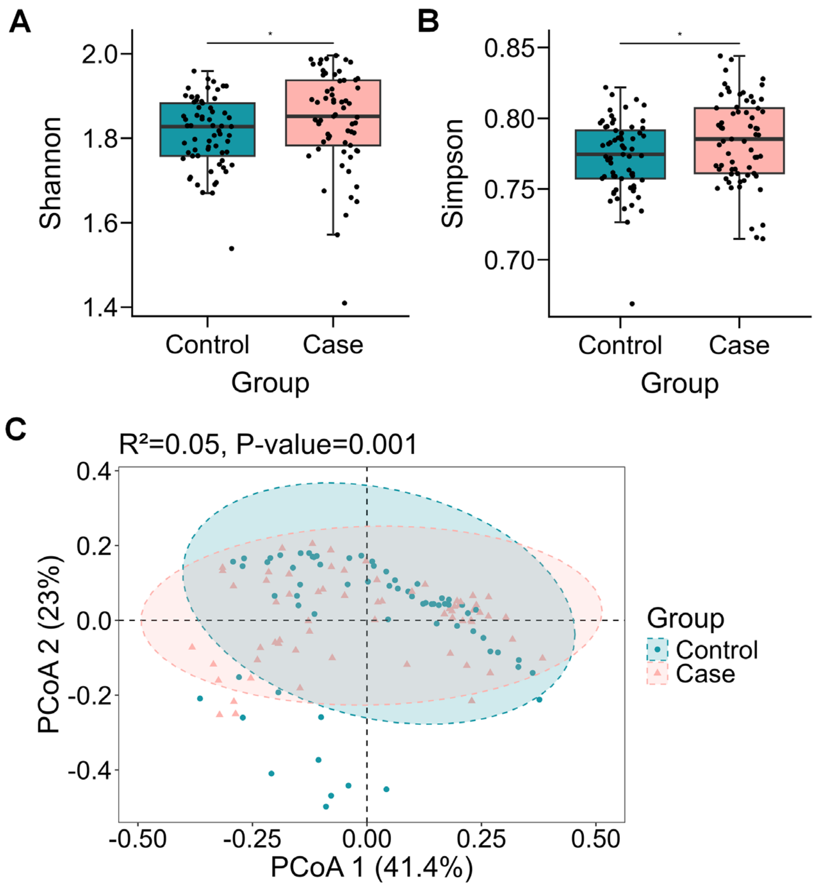

Supplementary Fig. 13 Differences in microbial community composition in the *C. difficile* infection dataset. **(A)** α-diversity analysis based on Shannon index. **(B)** α-diversity analysis based on Simpson index. **(C)** β-diversity analysis based on the Bray-Curtis distance. * means P-value < 0.05, ** means P-value < 0.01, and *** means P-value < 0.001.

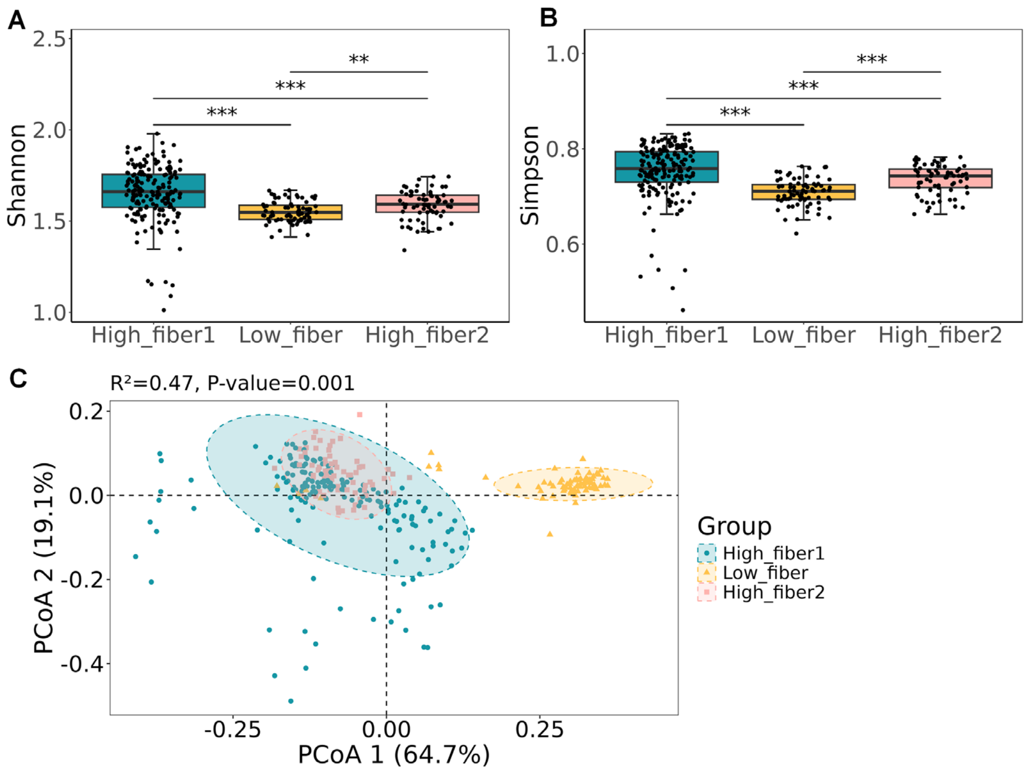

Supplementary Fig. 14 Differences in microbial community composition in the probiotic cocktail dataset. **(A)** α-diversity analysis based on Shannon index. **(B)** α-diversity analysis based on Simpson index. **(C)** β-diversity analysis based on the Bray-Curtis distance. * means P-value < 0.05, ** means P-value < 0.01, and *** means P-value < 0.001.

Supplementary Tables

Supplementary Table 1. Formal pairwise comparisons of interaction parameter estimation performance among sRidge, pNODE, and gNODE. See Supplementary Table 1 in Excel format.

Supplementary Table 2. Formal pairwise comparisons of trajectory prediction performance among NODE, pNODE, and gNODE. See Supplementary Table 2 in Excel format.

Supplementary Table 3. Formal pairwise comparisons of perturbation response prediction performance among NODE, pNODE, and gNODE. See Supplementary Table 3 in Excel format.

Supplementary Table 4. Empirical variability of inferred off-diagonal interaction coefficients across repeated gNODE refits.

**Time points**	**Mean empirical 95% interval width of off-diagonal β**	**Mean SD of off-diagonal β**
5	0.0527	0.0927
10	0.0452	0.0795
15	0.0461	0.0811
20	0.0469	0.0826
25	0.0483	0.0849

Supplementary Table 5. Degree metrics of strains under different dietary fiber conditions

Microbe	High_fiber	Low_fiber	High_fiber2
Strain4	11	7	8
Strain6	14	19	20
Strain7	8	7	9
Strain9	14	8	11
Strain13	19	17	16
Strain14	11	9	10
Strain15	13	12	10
Strain16	9	7	11
Strain21	5	10	8
Strain26	13	14	13
Strain27	6	6	6
Strain28	9	9	9
Strain29	10	17	11
